# Supplementary material for: Sleeping duration, physical activity, alcohol drinking and other risk factors as potential attributes of metabolic syndrome in adults in Ethiopia: A hospital-based cross-sectional study
Source: PLoS One. 2022 Aug 29;17(8):e0271962. doi: 10.1371/journal.pone.0271962 (PMC9423638; doi:10.1371/journal.pone.0271962)
Supplement: Attachment — Submitted filename: Evaluation Report - Manuscript Review Due 19 Feb 2022 PLOS ONE.docx [file pone.0271962.s001.docx]

# **EVALUATION REPORT**

## **Study Title**: Sleeping Duration, Physical Activity and Alcohol Drinking as Potential Attributes of Metabolic Syndrome in Adults in Ethiopia

## 1. **Summarise the main findings of the study and its purpose**

This manuscript is a herculean effort and an enjoyable read. The study’s objectives are essential in understanding the prevalence of metabolic syndrome and patterns of attributable risk factors in adults. This topic is significant, considering the more ageing adult population’s unprecedented growth and the projected increase in the prevalence of metabolic syndrome globally. Thus, mapping context-specific prevalence levels and attributable specific risk factors in adults is imperative.

Here, Mulugeta Belayneh *et al*., survey cross-sectionally Sleeping Duration, Physical Activity and Alcohol Drinking as Potential Attributes of Metabolic Syndrome in Adults in a single hospital in Ethiopia using the WHO-STEP-wise approach for non-communicable disease surveillance in developing countries.

## 2. **Highlights of the limitations and advantages**

There are many strengths to this study such as clear objectives, the focus on a specific population age group, detailed steps in the study’s design methodology, the uses of standard guidelines and definitions such as the WHO-STEP-wise approach for non-communicable disease surveillance in developing countries, the 2009 harmonised definition made by the International Diabetes Federation Task Force.

The study is well written. However, critical elements of cross-sectional research require additional attention concerning the reporting of the study and methodological details. I provide further explanations below.

## 3. **Factual errors or fundamental flaws? If yes, please detail your concerns**

I have offered specific comments below. Thus, improving the clarity of the study’s reporting guidelines will, in my mind, undoubtedly increase its impact. The study design methodology is currently missing crucial information that should be pre-specified comprehensively and as far as possible in the abstract and methods section:

### **TITLE & ABSTRACT**

==================

#### **Major comments:**

1) The authors have selected an essential topic for the study. As applicable in the title and abstract, they have included two important objectives, namely to measure the prevalence of the metabolic syndrome and to identify specific risk factors among adult populations in a single hospital in Ethiopia:
a) The authors need to provide an informative and balanced summary of what was done and findings in the abstract:

- The methods section is missing critical elements such as details on the primary variable of interest;
- Authors need to provide context and information about their primary measure. Within your Abstract Methods section, please include brief details on “Choice of primary measure”, an explanation of how the collected data on the analysis of the variable was performed, what statistical software was used, and what statistical tools were used to calculate prevalence or the measures of interest;
- Observational studies (cohort, case-control, or cross-sectional designs) must be reported in accordance with the STROBE statement; however, it is not specified in this section of the abstract which “reporting guidelines” informed this study, or whether it was communicated with study protocols; this information must be clearly indicated or should include a link to the full study protocol published; and
- To find reporting guidelines, see: <http://www.equatornetwork.org> and could the reporting checklist be included in the online supplementary material?

b) The abstract results section is unclear if the results reported here are “Before or After adjustment.” In this case, authors can simply state within the Abstract Results section by including “After or Before adjustment…”
c) Separate reporting of results by demographic variables, such as age and sex, facilitates pooling of prevalence for subgroups should be routine, unless inappropriate. I ask the authors to please report/ discuss the influence or association of variables, such as sex and/or gender, on their findings, where appropriate in the Abstract Section, Main Text and the limitations of the data in the main text.

- I would like to suggest that the authors consider “grouping the reporting of the identified specific risk factors” based on which were significantly associated with lower/ higher prevalence of the metabolic syndrome within gender. In my opinion, doing so would undoubtedly increase the study’s impact and make it more informative; and
- Alternatively, reporting on which of the remaining factors were positively/negatively associated with metabolic syndrome would be more appealing to inform the design of targeted intervention designs, thus supporting the recommendations made by the authors in the conclusion section.

d) Although the increased long-term risk of MetS in adults is known, it still is not something that doctors provide advice on at the visit in many settings. The authors could highlight the importance of their findings in affecting practice and policy in the Abstract conclusion section and main text to increase the report’s impact.

#### **Minor comment:**

1) Please check the document for consistent formatting of the text. It’s best to use consistent spacing between sentences within a document. This document should use: type a single space between words [Page 2; Line 37, 41 – 43];
2) Please indicate the study’s design with a commonly used term in the title also as it appears in the abstract “A hospital-based cross-sectional study…” [Page 2; Line 32];
3) Please correct the typo in the background section of the abstract “the metabolic syndrome had not to be investigated….” [Page 2; Line 29]; and
4) Please revise the conclusion in the abstract to avoid overly causal language.

### **INTRODUCTION**

==============

#### **Major comments:**

1) The rationale for undertaking this cross-sectional study in the context of already existing evidence has not been clearly explained:

- I am uncertain about the novelty of the research question and rationale for the investigation being reported. Lifestyle management is an established part of MetS prevention and treatment [the authors are aware and stipulated on Page 4; Line 88 – 89]. Also, the authors communicated that “Previous studies (dated 2016 and 2019) conducted in Ethiopia have documented a high prevalence of MetS” [Page 2; Line 83 – 84]. Please emphasise how the research question built up on the existing evidence; the reader needs to understand these links.

2) Please check the entire document for consistent formatting of the text. It’s best to use consistent spacing between sentences within a document. I suggest that this document should use: type a single space between words.
3) The last paragraph needs to have a statement about what is unknown and how this study adds to such knowledge [Page 2; Line 85 – 91].

### **METHODS**

========

#### **Major comments:**

1) Please specify what type of interview in the 1st paragraph [Page 4; Line 104 – 106].

### **DISCUSSION**

============

#### **Major comments:**

1) Please add a paragraph to start with, which should clearly state the most important findings and discuss what is novel.
2) Many paragraphs seem to repeat what is in the results. All paragraphs should have concluding statements stating what is new and how it is relevant for the field.
3) Discuss sources of limitations of the study, considering sources of potential bias or imprecision. Discuss both the direction and magnitude of any potential bias. The reader needs to understand the link between these.
4) To support the conclusion of this report, please give a cautious overall interpretation of results considering objectives, limitations, the multiplicity of analyses, results from similar studies, and other relevant evidence.
5) Discuss the study results’ generalisability (external validity), considering objectives and limitations of the study.
