# Supplementary material for: Sleeping duration, physical activity, alcohol drinking and other risk factors as potential attributes of metabolic syndrome in adults in Ethiopia: A hospital-based cross-sectional study
Source: PLoS One. 2022 Aug 29;17(8):e0271962. doi: 10.1371/journal.pone.0271962 (PMC9423638; doi:10.1371/journal.pone.0271962)
Supplement: Attachment — Submitted filename: Response to Reviewers.docx [file pone.0271962.s002.docx]

Response to Reviewers and Academic Editor

We the authors extremely acknowledge and appreciate all of the reviewers and the Academic Editor for their critical judgments and suggestions given to us. We have admitted and corrected our errors found throughout the manuscript according to the give comments and necessary modifications were made to enhance the quality of the paper. Changes were highlighted using track change. Our point-by-point responses are given below:

**Academic Editor**

- Use of the central obesity measure and cut-offs, clarify, and add additional analyses using different cut-offs.

Response: we have used optimal cut-off points recommended for Sub-Saharan countries i.e. 94 cm for men and 80 cm for women as recommended by IDF was used to define it.

- Identification of U-shaped relationships should be based on the multivariable analysis. Looking at the adjusted ORs no U-shape relationships were identified though. If there are any U-shape relationships, please present them graphically using log odds or log odds ratio plots for dose - response non-linear continuous exposure.

Response: we have corrected. It was our mistake that line graph was generated for the four exposure variables using their log odds ratio continuously so that upward curve at the beginning and end with many ups and downs in middle of the curve. Now we have revised it and present graphically for each exposure variable separately.

Thank you very much for your comment.

- Better describe choice and definition of risk factors; as well provide more details about the study sites.

Response: we tried to address the comments raised here and shown in red colour.

- Make sure to adhere to STROBE reporting guidelines for cross-section studies.

Response: we used the STROBE guideline to report the manuscript.

- Also note that reviewer 2 included the review of your paper as a separate Word document.

Response: we have also addressed the comments attached from reviewer 2.

Thank you so much for your critical comments and contributions.

**Reviewer #1**

We really appreciate the reviewer for giving such scholarly feedbacks that will improve visibility of the study. We thank you for your critical comments and time devotion.

 Q1. The title: should be reconsidered. Looking at the line 235 of the manuscript, several factors are associated with the metabolic syndrome .eg sex and age, yet the author chooses only a few for the title. They may consider "associated factors" without being specific.

Response to Q1: Yes, you are right and it is more inclusive if we change the title as “metabolic syndrome and associated factors”. But our concern is that when we see the strength of associations among these risky behavioural factors with MetS, it is stronger as compared to demographic factors and all this factors are amenable for change. They haven’t got attention as alternative prevention strategies for the growing burden of NCDs in developing nations including Ethiopia. We believe that this study will call for further scientific evidence and revision of existing policy.

Q2. Abstract: The conclusion on the abstract would need revision. in Line 44 the author uses the words " very emerging " which does not sound right given that we are not comparing it with any specific time in the past , to provide evidence of an increase.

Response to Q2: we admitted the comment and corrected.

Q3. Introduction: Would be useful to justify in a paragraph why the author chose a hospital population and not a community sample.

Response to Q3: Thank you for this important question. We selected the hospital population due two main issues: 1. to assure the precision and accuracy of biochemical analysis. 2. Due to financial limitation in community based study- costs are very expensive for field work from census, sample collection, transportation, and preservations procedure and prone for poor precision and validity.

Q4. Methods: 1. Line 101 need to be checked. The author says " simple random sample to catch up on the subjects" which does not sound very scientific.

Response to Q4.1: Yes it was the big challenge. To avoid non-probalistic sampling technique, we chose lottery method. What we did is that study subjects who fulfilled the inclusion criteria were identified and marked with red colour at the triage. Sampling frame using client’s card number had been prepared in morning of each data collection period. Then folding and rolling of papers carried out and the selected individuals linked to corresponding ward.

Q 4.2.Line 105: Why did the author choose WHO Steps survey, would help to describe this a bit more and provide reference.

Response to Q 4.2: Yes we tried to elaborate it in the method section. This surveillance is designed for both diagnosis of non-communicable diseases such as hypertension, diabetes, CVDs, and cancer and identification of risk factors. So it is an international tool and creates fair comparisons across regions.

Q4.3. Line 125: What is “Ethiopia’s adult classification System"
Define first terms the first time they appear, See FBD, HDL ( lines 131, 135).

Response to Q 4.3: it is now clarified more. We had used the international adult overweight and obesity cut-off points of BMI which was validated in western population (in US) before the development of the Ethiopian local standard in 2018 for classifying body weight. We used these optimal cut-off points to categorize BMI because the uses of the international cut-off points underestimate the prevalence of overweight and obesity. The logic behind is that presence of biological variations such as Ethiopian population are relatively shorter and slender. So an individual with a BMI of 23 is considered overweight. More details are found at our reference number 6. Sorry for clear mistakes in the use of abbreviations and now we corrected all.

Q 4.4. Line 141 Give reference for General physical Questionnaire and report if it has been used previously in similar settings.

Response to Q 4.4: thank you so much, it is corrected (supported with reference 40).

Q 4.5. Line 156: Give reference for the food questionnaire

Response to Q 4.5: we used reference number 30 and 31 for food frequency questionnaire to assess dietary habits of the study subject.

Q 4.6: Line 163: Justify the fact that the one question on sleep is adequate.

Response to Q 4.6: we tried to justify it but lack of internationally validated tool affects comparability of the findings. We categorized sleep duration as recommended by the National Sleep foundation of America for the regression analysis.

Q 5. Discussion: include limitations of the study.

Response to Q5: Thank you for your critical comment and we added the limitations of the study.

**Reviewer #2**

It’s our heartfelt gratitude to acknowledge the reviewer for his/her unreserved and extensive outlooks for enhancing the quality of this flawed manuscript. Your critical questions and suggestions really helped us to thoroughly modify the manuscript accordingly.

## **Q 1. Factual errors or fundamental flaws? If yes, please detail your concerns**

I have offered specific comments below. Thus, improving the clarity of the study’s reporting guidelines will, in my mind, undoubtedly increase its impact. The study design methodology is currently missing crucial information that should be pre-specified comprehensively and as far as possible in the abstract and methods section:

### **TITLE & ABSTRACT**

==================

#### **Major comments:**

1) The authors have selected an essential topic for the study. As applicable in the title and abstract, they have included two important objectives, namely to measure the prevalence of the metabolic syndrome and to identify specific risk factors among adult populations in a single hospital in Ethiopia:
a) The authors need to provide an informative and balanced summary of what was done and findings in the abstract:

- The methods section is missing critical elements such as details on the primary variable of interest;
- Authors need to provide context and information about their primary measure. Within your Abstract Methods section, please include brief details on “Choice of primary measure”, an explanation of how the collected data on the analysis of the variable was performed, what statistical software was used, and what statistical tools were used to calculate prevalence or the measures of interest;
- Observational studies (cohort, case-control, or cross-sectional designs) must be reported in accordance with the STROBE statement; however, it is not specified in this section of the abstract which “reporting guidelines” informed this study, or whether it was communicated with study protocols; this information must be clearly indicated or should include a link to the full study protocol published; and
- To find reporting guidelines, see: <http://www.equatornetwork.org> and could the reporting checklist be included in the online supplementary material?

Response to 1a: Thank you for your important comments. We did it accordingly and modifications are highlighted through track changes

b) The abstract results section is unclear if the results reported here are “Before or After adjustment.” In this case, authors can simply state within the Abstract Results section by including “After or Before adjustment…”

Response 1 b: it right and corrected.

c) Separate reporting of results by demographic variables, such as age and sex, facilitates pooling of prevalence for subgroups should be routine, unless inappropriate. I ask the authors to please report/ discuss the influence or association of variables, such as sex and/or gender, on their findings, where appropriate in the Abstract Section, Main Text and the limitations of the data in the main text.

- I would like to suggest that the authors consider “grouping the reporting of the identified specific risk factors” based on which were significantly associated with lower/ higher prevalence of the metabolic syndrome within gender. In my opinion, doing so would undoubtedly increase the study’s impact and make it more informative; and
- Alternatively, reporting on which of the remaining factors were positively/negatively associated with metabolic syndrome would be more appealing to inform the design of targeted intervention designs, thus supporting the recommendations made by the authors in the conclusion section.

Response 1 c: we tried to address the above comments. In this revision we try to report the findings by stratifying metabolic syndrome by gender and other demographic variables in abstract, result and discussion sections.

d) Although the increased long-term risk of MetS in adults is known, it still is not something that doctors provide advice on at the visit in many settings. The authors could highlight the importance of their findings in affecting practice and policy in the Abstract conclusion section and main text to increase the report’s impact.

Response to 1 d: we accepted the comment and incorporated.

#### **Minor comment:**

1) Please check the document for consistent formatting of the text. It’s best to use consistent spacing between sentences within a document. This document should use: type a single space between words [Page 2; Line 37, 41 – 43];

Response to Q 1: thank you and it’s corrected.

2) Please indicate the study’s design with a commonly used term in the title also as it appears in the abstract “A hospital-based cross-sectional study…” [Page 2; Line 32];

Response to Q 2: Edited.

3) Please correct the typo in the background section of the abstract “the metabolic syndrome had not to be investigated….” [Page 2; Line 29]; and

Response to Q3: Thanks and edited.

4) Please revise the conclusion in the abstract to avoid overly causal language.

Response to Q 4: we tried to modify using track changes. Thank you.

### **INTRODUCTION**

==============

#### **Major comments:**

1) The rationale for undertaking this cross-sectional study in the context of already existing evidence has not been clearly explained:

- I am uncertain about the novelty of the research question and rationale for the investigation being reported. Lifestyle management is an established part of MetS prevention and treatment [the authors are aware and stipulated on Page 4; Line 88 – 89]. Also, the authors communicated that “Previous studies (dated 2016 and 2019) conducted in Ethiopia have documented a high prevalence of MetS” [Page 2; Line 83 – 84]. Please emphasise how the research question built up on the existing evidence; the reader needs to understand these links.

Response to Q 1: we tried to justify the necessity of the current study in Ethiopia in introduction section. The first issue was the presence of variations in the use of criteria for diagnosing MetS. Some use the National Cholesterol Education program criteria. Second, presence of unclear methodological approach how they were measured biomedical tests, measurements related to physical activity, anthropometrics measures, and some other behavioural factors. Lastly, the previous studies hadn’t addressed sleeping duration and dietary habits in particular consumption of fruits, vegetables and unhealthy foods.

2) Please check the entire document for consistent formatting of the text. It’s best to use consistent spacing between sentences within a document. I suggest that this document should use: type a single space between words.

Response to Q 2: We have edited the whole document.

3) The last paragraph needs to have a statement about what is unknown and how this study adds to such knowledge [Page 2; Line 85 – 91].

Response to Q 3: We modified accordingly.

**Methods:**

#### **Major comments:**

1) Please specify what type of interview in the 1st paragraph [Page 4; Line 104 – 106].

*Response to Q1: it is now specified.*

### **DISCUSSION**

============

#### **Major comments:**

1) Please add a paragraph to start with, which should clearly state the most important findings and discuss what is novel.

Response Q 1: we have added the main findings of the study and discussed

2) Many paragraphs seem to repeat what is in the results. All paragraphs should have concluding statements stating what is new and how it is relevant for the field.

Response Q 2: we tried to revise the discussion section and some amendments were taken.

3) Discuss sources of limitations of the study, considering sources of potential bias or imprecision. Discuss both the direction and magnitude of any potential bias. The reader needs to understand the link between these.

Response to Q 3: we have added the limitations that may exist due to an avoidable bias and technical gaps that we have address in the study.

4) To support the conclusion of this report, please give a cautious overall interpretation of results considering objectives, limitations, the multiplicity of analyses, results from similar studies, and other relevant evidence.

Response 4: We have tried to address these issues in the last paragraphs of discussion.

5) Discuss the study results’ generalizability (external validity), considering objectives and limitations of the study.

Response to Q 5: we have also mentioned it the generalizability of the study and possible research perspectives.

Thank you so much.

**Reviewer #3**

We thank you very much for the critical and constructive comments and suggestion given. We tried to address the comments here below:

Q 1. Abstract: line 45 - " U " shaped relationships. Relationship for e.g between physical activity and MetS is not U shaped. Low, moderate and high physical activity - The prevalence of Mets is 45%, 27.5% and 13.2 % respectively. Similarly with sleep duration and education level

Response to Q 1: Thank for your critical comment. Actually our concern was to describe the overall relationships of predictor variables and MetS. While observing the directions associations, all variables have positive beta-coefficients except no formal education which has negative beta-coefficient. We assume that outcome (MetS) a multiplicative result of a positive β (slope) of physical activity, age, sex, sleep duration, residence and alcohol consumption and a negative β of no formal education which will cross the other factors at somewhere. This was our mistake and we have corrected.

2. Inconsistent use of waist circumference cut -off values. Line 63-64 - 83.7 cm and 78 cm for males and females respectively - Authors use country specific WC cut off. However, Line 126- 127 , - Authors use IDF criteria 94cm and 80 cm respectively for males and females

Response to Q 2: Thank you for your comments. We believed that there should be further clarity in the introduction part and now we have added additional description. The cut-off points for 83.7 and 78 cm for men and women recommended by the WHO and World heart Federation to define higher WC. This was used to diagnosis MetS before the harmonized criteria made by IDF become available in 2009 and after onward central obesity is defined when WC is greater than 94cm and 80 cm in men and women respectively. So we used this cut-off point to measure central obesity.

3. Sleep categories inconsistent and incorrectly described. Lines 164 -165 - 4 categories noted, However only 3 are described. Furthermore, the categories in Table 2 are not consistent with those described in the method (lines 164 -165 ), or in the publication that the authors use as a reference

Response to Q 3: Thank you again for the comment. The sleep duration data were collected using pre-categorised groups as < 6 hrs, 6 to 7 hrs, 8 to 9 hrs, and 10 hrs and above. These are the four categories presented in the Table 2 of result section. But we merged responses found in the second and third category during the regression analysis (Table 4) considering the recommended healthy sleep duration range 6 to 9 hrs by National Sleep Foundation of CDC.

4. Line 169 - Variables with a p value of less than 0.2 in the bivariate analysis were exported to the final model. Although I am aware that it is not incorrect to use this level of significance , can the authors please explain the rationale behind the use of this level of significance in this study.

Response Q 4: This is by consensus that increasing the default removal value of α from 0.1 to 0.2 will allow chance for some clinically important variables or previous other empirical parameters to be iterated in the multiple regression models. If we change the value to 0.2, variables with p-value b/n 0.1 to 0.2 in the bivariate analysis will have chance in the final model and may become significant when confounding controlled. Unfortunately recent softwares have power to handle such issue at the default setting of 0.1 and may not be necessary. Even we didn’t find a variable with p-value >0.1 in the bivariate that is significant in the multivariable analysis.

5. Line 256 -this statement is not correct in two respects.
a. The results do not support this statement. The authors state that there was an inverse relationship between level of education and MetS. However, in Table 2 the prevalence of MetS is 20.1 % ,33.5% and 41.9 % in patients with no education, a high school level of education and a college education respectively

Response Q 5 a: These percentages don’t tell us the presence of relationships rather odds ratio reflects whether a certain factor is a preventive or a risk for occurrence of MetS. In actual scenario more educated persons believed to have adequate knowledge and skills to prevent themselves from health problem. In our case, educated individuals are more prone to MetS than those who had no education. We have edited that section and modifications are included.

b. The publication referenced does not contradict this statement. Santos et al (reference 48) indeed reported an inverse relationship between educational level and the prevalence of MetS in their study

Response Q5 b: According to reference 48, the odds of MetS significantly decrease as the level of education increases. The opposite is true in the case of our finding.

6. Table 1 -No currency for monthly income level categories

Response to Q6: we have edited.

7. Table 2 - Poorly formatted.

Response Q 7: we formatted again.

8. Table 3 - "Consumption outside home” needs to be clarified.

Response to Q 8: we tried to elaborate it by adding consumption in cafeteria, restaurant or in hotel.

9. Table 5 - The prevalence of MetS should be presented as n(%) ,not only n

Response Q 9: we have done it.

10. An examples of a significant language error

lines 287- 289 : "However, the clear underlying mechanisms related to the relationship between shorter sleep duration and MetS are understood."
I believe the authors meant to say the underlying mechanisms between duration of sleep and MetS are not clear or well understood

Response to Q 10: we have modified accordingly.

We extremely appreciate your contributions and observations.

**Reviewer #4**

We thank you very much for the critical and constructive comments and suggestion given. We tried to address the comments here below:

Reviewer #4: This is a very important topic area, metabolic syndrome and its associated risk factors are required in SSA population. However, authors should consider the following suggestions.

1. I think the current title over exaggerates the study, because other risk factors of metabolic syndrome were looked at in the study, why is the author focusing on sleep duration, alcohol drinking and physical activity in the title.

Response to Q1: Yes, you are right and it is more inclusive if we change the title as “metabolic syndrome and associated factors”. But our concern is that when we see the strength of associations among these risky behavioural factors with MetS, it is stronger as compared to demographic factors and all this factors are amenable for change. They haven’t got attention as alternative prevention strategies for the growing burden of NCDs in developing nations including Ethiopia. We believe that this study will call for further scientific evidence and revision of existing policy.

2. Line 206, the author referenced table 4 twice, Table Four ….., S, and BP (Table 4). Please reword

Response to Q 2: Thank you. We have made total modification. We removed table 4 in general since it’s not related to the objectives of the study.

3. The central obesity parameter that was used in this manuscript for African population might have over estimated or underestimated the prevalence among your population. The author have used .. waist circumference ≥83.7 64 cm for males and ≥78.0 cm for females. The harmonized definition suggested that 94 and 80cm should be used for SSA population (<https://www.ahajournals.org/doi/10.1161/CIRCULATIONAHA.109.192644?url_ver=Z39.88-2003&rfr_id=ori:rid:crossref.org&rfr_dat=cr_pub%20%200pubmed>) . Also an optimal waist circumference cut-off for SSA population has been published by Ekoru et al, 2018(<https://www.nature.com/articles/ijo2017240>). I will suggest that the authors should re-analyse using either of this cut off.

Response to Q 3: We used 94cm for men and 80 cm for women for WC to define central obesity as specified in method section. The introduction section was not clear in the manuscript but now it is clearer. 83.7 cm and 78 cm of WC were the old criteria to diagnose MetS and we performed it with harmonized criteria as recommended by ID.

4. Line 214 .. Aas one becomes aged the probability of having MetS increased as well. Please reword this sentence

Response Q 4: we have corrected it.

5. Generally, I will suggest that the Author edits the manuscript or engage an editor to complete that role on their behalf.

Response to Q 5: We have edited the manuscript.

6. Line 287 – 289 .. It seems that both the genetic and environmental factors causing MetS but 288 in this study, the environmental factors predominantly contributed to the rapid increment of 289 MetS and are fully subjected to modification given large-scale interventions are designed. I will suggest that the author should conclude the study following the focus of the variables that were collected, which in this instance are behavioural factors. The author has not collected r discussed any environmental factors for this study, so should not conclude

Response Q 6: accepted the comment and modified. Thank you very much for the overall constructive and critical comments.

**Reviewer #5**

We thank you very much for the critical and constructive comments and suggestion given. We tried to address the comments here below:

Reviewer #5: The metabolic syndrome is an increased public health issue particularly in low- and middle-income countries (as authors noted). It needs to be well described in both general population and specific population (like patients). The study is of important for both clinician and public health managers in Ethiopia to address MetS in all context.

Major comments
• Authors have noted (line 83-84) that “Previous studies conducted in Ethiopia have documented a high prevalence of MetS ». Thus, it is not clear why the study was conducted and which gap of knowledge it filled.

Response to Q1: we tried to justify the necessity of the current study in Ethiopia in introduction section. The first issue was the presence of variations in the use of criteria for diagnosing MetS. Some use the National Cholesterol Education program criteria. Second, presence of unclear methodological approach how they were measured biomedical tests, measurements related to physical activity, anthropometrics measures, and some other behavioural factors. Lastly, the previous studies hadn’t addressed sleeping duration and dietary habits in particular consumption of fruits, vegetables and unhealthy foods.

• Line 94-95: Study site was not described. Authors have to provide more details about the study site (is it a secondary of tertiary hospital? how many bed?, which department of hospital was targeted and why? How many outpatients attended by year? the hospital is located in rural or urban areas, etc.…)

Response to Q 2: we included background information about the study setting.
• Line 99: it is not clear how the sample size was calculated and how the simple ramdom sampling was performed how the study design was considered in the data analysis. Can authors provide more information about this?

Response to Q3: Sample size was determined using single proportion population formula by considering the prevalence of MetS as 45.3% from a study in Ethiopia, confidence level of 95%, 5% of margin of error and with 10% of non-response rate, which finally gave 419 study subjects. We had also determined the sample using Epi-Info for identifying factors of MetS to confirm that the sample is adequately powered. Finally the maximum sample was taken for the study. To avoid non-probalistic sampling technique, we chose lottery method. What we did is that study subjects who fulfilled the inclusion criteria were identified and marked with red colour at the triage. Sampling frame using client’s card number had been prepared in morning of each data collection period. Then folding and rolling of papers carried out and the selected individuals linked to corresponding ward.

• I suggest that authors improve the presentation of the results of their study, mainly subsection about the MetS (line 198-207). Indeed, they have to describe the prevalence of MetS in a paragraph and the components of MetS in another paragraph. In addition, It is not clear why authors check for a correlation between WC and others MetS components since this was not cited as study objective. I recommend the authors to be focus on their study objective in the presentation of the results of their study.

Response Q 4: we removed this section.
• Authors have to provide limitations of their study since the study has many limitations. First, it was conducted in hospital and may not be considered to make inference to the general population of Ethiopia. The behavioral variables might be affected by social desirability bias.

Response Q 5: We made modifications accordingly.
• Authors has to provide more details of public health or clinical pratice implication of their study since it was done in health facility. e.g: to organize sensitization campaign targeting patients attended in hospital.

Response to Q 6: we tried to address the issues.
Minors comments
• I suggest that authors in the title of the manuscript precise the type of the study

Response: accepted.
• Line 56: Please, correct the abbreviation of metabolic syndrome “MetS” instead of “Mets”

Response: corrected.
• Line 63: Please, precise which definition of metabolic syndrome was provided here. It is not clear in the text.

Response: we have clearly indicated in the method section now. We used the harmonized diagnosis criteria for MetS recommended by IDF.
• Line 68-69: authors had to indicate why they used the most recent definition (since it is also discussed)

Response: in the recent definition, central obesity is a must and any two of the four criteria is used to measure MetS but the former criteria to diagnose MetS was the presence of at least three of the five components was used.
• line 73 to 75: Please, provide the reference of the statement

Response: corrected.
• Line 133: the mean of (26) is not clear.

Response: it is edited.
• Line 172: the acronym AOR was use for the first, it needs to be defined.

Response: corrected.
• Line 186-190: It is not clear if the prevalence of MetS reported in this paragraph is the history of MetS or MetS diagnosis during the current study. Please, can authors provide clarification?

Response: It is from currently diagnosed subjects.
• Line 214: correct Aas

Response: corrected.
• Table 3 line 1 : Please the number 3(0.7)

Response: it is corrected.
• Please improve the tables formatti

Response: we have formatted the tables accordingly.

Thank you very much for significant contributions and critical comments given to us.
