# Supplementary material for: Sleeping duration, physical activity, alcohol drinking and other risk factors as potential attributes of metabolic syndrome in adults in Ethiopia: A hospital-based cross-sectional study
Source: PLoS One. 2022 Aug 29;17(8):e0271962. doi: 10.1371/journal.pone.0271962 (PMC9423638; doi:10.1371/journal.pone.0271962)
Supplement: Attachment — Submitted filename: METS SSTUDY COMMENTS.docx [file pone.0271962.s003.docx]

Additional METS STUDY COMMENTS

1. I still feel the Title needs to be reflective of the content of the manuscript. Because the authors accepts that fact, they should coin a title that includes more than sleep, physical activity and alcohol.
2. Given that only one question was asked about sleep, I propose that this is captured as part of the limitations.
3. I appreciate the inclusion of the limitation section-the statement “ undoubtedly reproducible” on line 345 can be replace with more gentle statement like“ may be generalized..because….”
4. The last two sentences on the conlcuion section	do not sound appropriate	They can be reworded to encourage not sound like a warning “Unless the national health policy for NCDs prevention is revised and effectively implemented, the developmental progress of the nation will be stepped backed due to the high health care expenditure, high disability adjusted life years and economic crisis. In general the global commitment designed targets in the Sustainable Development Goals will be off-track by 2030”
